# AD−1 Small Molecule Improves Learning and Memory Function in Scopolamine-Induced Amnesic Mice Model through Regulation of CREB/BDNF and NF-κB/MAPK Signaling Pathway

**DOI:** 10.3390/antiox12030648

**Published:** 2023-03-05

**Authors:** Rengasamy Balakrishnan, Ju-Young Park, Duk-Yeon Cho, Jae-Yong Ahn, Dong-Sun Yoo, Sang-Ho Seol, Sung-Hwa Yoon, Dong-Kug Choi

**Affiliations:** 1 Department of Applied Life Science, Graduate School, BK21 Program, Konkuk University, Chungju 27478, Republic of Korea; 2 Department of Biotechnology, College of Biomedical and Health Science, Research Institute of Inflammatory Disease (RID), Konkuk University, Chungju 27478, Republic of Korea; 3 Department of Molecular Science and Technology, Ajou University, Suwon 16499, Republic of Korea; 4 Research and Development, Sinil Pharmaceutical Co., Ltd., & APIMEDS Inc. Room 608 Namseong Plaza Building, Digital-ro 130 Geumcheon-gu, Seoul 08589, Republic of Korea

**Keywords:** AD−1 small molecule, scopolamine, oxidative stress, memory impairment, neuroinflammation, apoptosis

## Abstract

Cognitive decline and memory impairment induced by oxidative brain damage are the critical pathological hallmarks of Alzheimer’s disease (AD). Based on the potential neuroprotective effects of AD−1 small molecule, we here explored the possible underlying mechanisms of the protective effect of AD-1 small molecule against scopolamine-induced oxidative stress, neuroinflammation, and neuronal apoptosis. According to our findings, scopolamine administration resulted in increased AChE activity, MDA levels, and decreased antioxidant enzymes, as well as the downregulation of the antioxidant response proteins of Nrf2 and HO-1 expression; however, treatment with AD−1 small molecule mitigated the generation of oxidant factors while restoring the antioxidant enzymes status, in addition to improving antioxidant protein levels. Similarly, AD−1 small molecule significantly increased the protein expression of neuroprotective markers such as BDNF and CREB and promoted memory processes in scopolamine-induced mice. Western blot analysis showed that AD−1 small molecule reduced activated microglia and astrocytes via the attenuation of iba-1 and GFAP protein expression. We also found that scopolamine enhanced the phosphorylation of NF-κB/MAPK signaling and, conversely, that AD−1 small molecule significantly inhibited the phosphorylation of NF-κB/MAPK signaling in the brain regions of hippocampus and cortex. We further found that scopolamine promoted neuronal loss by inducing Bax and caspase-3 and reducing the levels of the antiapoptotic protein Bcl-2. In contrast, AD−1 small molecule significantly decreased the levels of apoptotic markers and increased neuronal survival. Furthermore, AD−1 small molecule ameliorated scopolamine-induced impairments in spatial learning behavior and memory formation. These findings revealed that AD−1 small molecule attenuated scopolamine-induced cognitive and memory dysfunction by ameliorating AChE activity, oxidative brain damage, neuroinflammation, and neuronal apoptosis.

## 1. Introduction

Alzheimer’s disease (AD) is a highly prevalent neurodegenerative disorder, and the number of patients gradually increases with the passage of time. AD is characterized by progressive cognitive impairment, memory loss, and personality changes due to neurodegeneration in the hippocampus and frontal cortex [1,2]. The main etiology of AD has yet to be elucidated definitively. The pathological hallmarks of AD include the accumulation of senile plaques, neurofibrillary tangles, and cholinergic neuronal degeneration [3]. AD pathology also includes oxidative stress, neuroinflammation, and neuronal death, which consequently leads to memory impairment and cognitive dysfunction [4]. Cholinergic system dysfunction causes a decline in acetylcholine (Ach) levels and plays a critical role in the pathogenesis of AD [5]. These cognitive impairments may be caused by changes in morphology, such as structural changes in neurons, synapses, and nerve fibers; changes in neurotransmitter levels; or oxidative brain damage [6].

Scopolamine is known as a pharmacological model of memory loss that acts by inhibiting the muscarinic acetylcholine receptor, which is an important component of the cholinergic system. The systemic administration of scopolamine induces oxidative stress, mitochondrial dysfunction, neuroinflammation, and neuronal apoptosis and an increase in acetylcholinesterase (AChE) activity [7,8]. A clinical study demonstrated that scopolamine modulates the locomotor activity and impairs hippocampal-dependent spatial learning and memory formation by impeding cholinergic neurotransmission [9,10]. Interestingly, interrupted cholinergic activity is also accompanied by increased levels of oxidative stress [11]. More specifically, scopolamine was shown to reduce the activity of antioxidant enzymes, while also increasing the level of lipid peroxidation in the cortex and hippocampus of AD mice [12]. Nuclear factor erythroid two-like 2 (Nrf2) is a transcription factor that regulates heme oxygenase-1 (HO-1) expression, an important antioxidant enzyme that plays a major role in neutralizing oxidative stress. However, some studies have indicated that scopolamine induced the downregulation of Nrf2/HO-1 also involved in AD pathology [13,14]. Furthermore, there is increasing evidence that scopolamine inhibits with the molecular homeostasis of neuroprotective or defensive proteins CREB/BDNF in animal models [14]. Despite this, the majority of studies have demonstrated the expression and release of various inflammatory factors closely linked to the downstream activation of NF-κB and p38 MAPK signaling pathways, which are subsequently involved in memory processes, which has been reported elsewhere [15,16].

**Figure 1:**
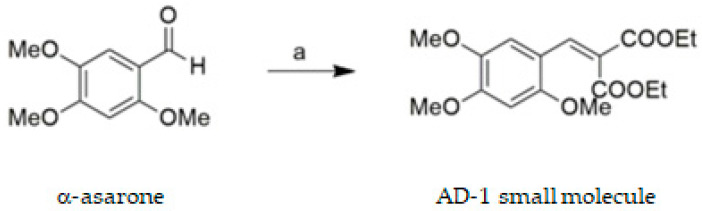
Chemical structure of α-asarone and AD−1 small molecule.

In the present study, we synthesized diethyl 2-(2,4,5-trimethoxybenzylidene) malonate (AD−1 small molecule), an analogue from α-asarone which has long been used as a neuroprotective medicine. They play multiple biological roles and have been confirmed to be a significantly neuroprotective agent against cognitive and memory dysfunction in animal models [17,18]. Recent reports have shown that α-asarone might exert anti-inflammatory effects and, subsequently, ameliorate Parkinson’s by improving neuronal survival, as well as neurotransmitter levels [19]. Therefore, these existing results encouraged us to perform a series of experiments to evaluate the anti-amnesic effects of AD−1 small molecule. Here, we investigated the neuroprotective and anti-amnesic effects of AD−1 small molecule on scopolamine-induced cognitive impairments in mice. Moreover, we examined whether the memory-ameliorating effects of AD−1 small molecule were associated with oxidative stress and neuroinflammation. The chemical structure of α-asarone and AD−1 small molecule is presented in Figure 1.

## 2. Materials and Methods

### 2.1. Chemicals and Reagents

Scopolamine, tacrine, and methylcellulose were procured from Sigma-Aldrich (St. Louis, MO, USA). RIPA lysis buffer (10X) was acquired from Millipore (Milford, MA, USA). A phosphatase and protease inhibitors cocktail was obtained from Roche (Indianapolis, IN, USA). Tween 80 was acquired from Calbiochem (Gibbstown, NJ, USA). Anti-iNOS (at 135 kDa; Abcam, ab15323), anti-COX-2 (at 69 kDa; Abcam, ab15191), anti-iba-1 (at 17 kDa; Abcam, 153696), anti-BDNF (at 15, 28 kDa; Abcam, ab108319), and anti-Nrf-2 (at 110 kDa; Abcam, ab137550) antibodies were purchased from Abcam (Cambridge, United Kingdom). Anti-HO-1 (at 28 kDa; Cell signaling, #70081), anti-IκB-α (at 39 kDa; Cell signaling, #4812S), anti-p38 (at 40 kDa, Cell signaling, #9212S), anti-ERK (at 42, 44 kDa; Cell signaling, #9102S), anti-JNK (at 46, 54 kDa; Cell signaling, #9252S), anti-NF-κB (at 65 kDa; Cell signaling, 8242S), anti-p-IκB-α (at 65 kDa; Cell signaling, 9241S), anti-p-p38 (at 43 kDa; Cell signaling, 9211S), anti-p-ERK (at 42, 44 kDa; Cell signaling, 9101S), anti-p-JNK (at 46, 54 kDa; Cell signaling, 9251S), anti-p-NF-κB (at 65 kDa; Cell signaling, 3033S), and anti-β-actin antibodies were obtained from Cell Signaling Technology (Danvers, MA, USA). Anti-caspase-3 (at 19 kDa; Santa Cruz Biotechnology, #H-277), anti-Bcl2 (at 28 kDa; Santa Cruz Biotechnology, #N-19), and Bax (at 23 kDa; Santa Cruz Biotechnology, CD150) antibodies were purchased from Santa Cruz Biotechnology (Santa Cruz, CA, USA). Secondary antibodies conjugated with horseradish peroxidase (HRP) were procured from Sigma Aldrich (St. Louis, MO, USA). Biochemical assays were performed using commercially available kits. All other chemicals used in these experiments were provided by local commercial sources (analytical grade quality).

### 2.2. Synthesis of AD−1 Small Molecule

The synthesis of AD−1 small molecule was prepared by reacting commercially available 3,4,5-trimethoxybenzaldehyde with diethyl malonate via Knoevenagel condensation. Briefly, to the solution of 2,4,5-trimethoxybenzaldehyde (2.00 g, 10.2 mmol) in toluene (100 mL) was added diethyl malonate (1.64 mL, 10.8 mmol), piperidine (156 mg, 1.83 mmol), and acetic acid (306 mg, 5.10 mmol). The solution was stirred at a reflux temperature for 18 h. After the reaction mixture was evaporated in vacuo, the residue was purified by silica gel column chromatography to prepare AD−1 small molecule as a pale-yellow solid. (2.1 g, 60.9%) ^1^H NMR (600 MHz, CDCl_3_) δ 1.27 (dt, J = 17.2, 7.2 Hz, 6H), 3.76 (s, 3H), 3.82 (s, 3H), 3.89 (s, 3H), 4.28–4.23 (m, 4H), 6.45 (s, 1H), 6.97 (s, 1H), 8.02 (s, 1H); ^13^C NMR (151 MHz, CDCl_3_) δ 13.9, 14.1, 55.9, 56.2, 56.3, 61.2, 61.3, 96.4, 111.7, 113.4, 123.2, 137.1, 142.9, 152.5, 154.1, 164.7, 167.5.

### 2.3. Animals Handling and Treatment

Male C57BL/6 mice (age = 8 weeks old, weight = 20−25 g, *n* = 10) were purchased from Daehan Bio-Link, Korea. All animals were acclimatized for 7 days: they were housed in a controlled environment with lighting (12/12 h dark/light cycle) and were allowed free access to food and water. The animal experiments were approved by the Institutional Animal Care and Use Committee (IACUC) of Konkuk University (IACUC no. KU19112, KU23024).

**Figure 2:**
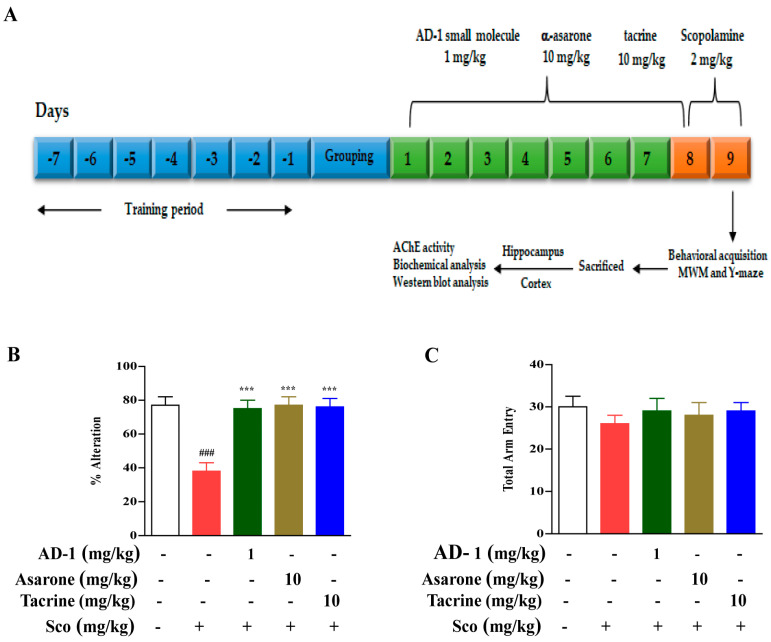

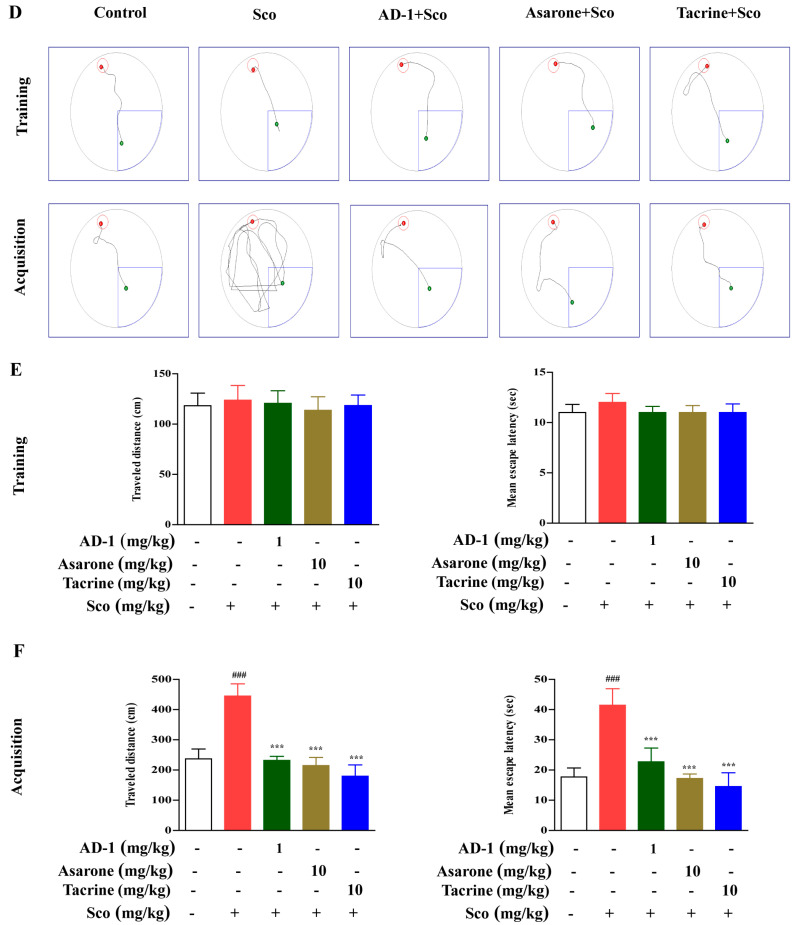
Effects of AD−1 small molecule on scopolamine-induced cognitive dysfunction by the Y-maze and MWM test. The overall experimental schedule performed in the present study shown in (**A**). Number of total arm entry (**B**) and spontaneous percentage of alterations (**C**). The trajectories show the path length covered by mice during the probe test (**D**). Distance travelled and escape latency to find a hidden goal training (**E**) and acquisition (**F**). Data are expressed as mean ± SD (n = 6 per group). ^###^ *p* < 0.001 scopolamine-treated group versus control; *** *p* < 0.001 group treated with AD−1 small molecule, asarone, and tacrine/scopolamine compared to group treated with scopolamine alone.

A preliminary dose selection experiment was performed to decipher the neuroprotective effect of various AD−1 small molecule doses on mice exposed to 1-methyl-4-phenyl-1,2,3,6-tetrahydropyridine (MPTP) (data not published). Based on the results from the preliminary dose-response experiment, 1 mg/kg/b.w. was selected for the pharmacological dose of AD−1 small molecule for further experiments. The doses of α-asarone and tacrine were chosen in accordance with our recently published research articles [19]. The animals were randomly divided into the following groups: group I, control; group II, scopolamine (2 mg/kg/b.w.); group III, AD−1 small molecule (1 mg/kg/b.w.) + scopolamine (2 mg/kg/b.w.); group IV, α-asarone (10 mg/kg/b.w.) + scopolamine (2 mg/kg/b.w.); and group V, tacrine (10 mg/kg/b.w.) + scopolamine (2 mg/kg/b.w.), which were considered a positive control. Scopolamine (2 mg/kg/b.w.) and tacrine (10 mg/kg/b.w.) was dissolved in saline, while AD−1 small molecule (1 mg/kg/b.w.) and α-asarone (10 mg/kg/b.w.) was dissolved in the physiological level of methylcellulose (0.5%) solution containing 1% Tween 80. Animals who received scopolamine (2 mg/kg/b.w. i.p.) were injected twice at 24 h intervals for a scopolamine scheduled day; the AD−1 small molecule, α-asarone, and tacrine treatment (p.o.) was administered once daily for 1 week. Their behavioral parameters, such as Y-maze and Morris water maze (MWM), were evaluated 30 min after the intraperitoneal injection of scopolamine. Following a behavioral assessment, the mice were anesthetized via an intraperitoneal injection of 23% urethan, as approved by the Ethical Committee of Konkuk University. Upon cessation of the heartbeat and breathing of the mice, the skull was removed, and the hippocampus and cortex were collected from the brain regions and stored at −80°C for biochemical, neurochemical, and Western blot analyses. The timeline of the experimental treatments conducted in this study is illustrated in Figure 2A.

### 2.4. Y-Maze Test

Y-maze tasks were used for the spatial and reference memory assessments. A “Y”-shaped maze with 3 identical arms—with each arm separated by a 120-degree angle—was used for the test. The mice were placed at the center of the maze, and the latency of their movement and altered entry into different arms were noted for 5 min. Mice with an intact memory were expected to show less interest in revisiting recently entered arms, while those with an impaired memory were more likely to revisit said arms [20].

### 2.5. Morris Water Maze Test

The MWM test was used to evaluate the spatial reference learning and memory of the mice. In general, the mice must rely on their navigational skills to detect the submerged escape platform. Here, a large circular pool (diameter = 122 cm; height = 35 cm) with a hidden platform was used. The MWM tank was filled with normal water to a depth of 20 cm and maintained at a temperature of 25 ± 2°C. Ink was added to the water to prevent the mice from seeing the platform. The animals were assessed on their ability to find a short and direct path to the hidden platform from their starting position. We used two back-to-back training sessions with four trials per day for three consecutive days before treatment. During these three consecutive days, the mice were allowed to rest on the platform for 10 s and were required to successfully locate the platform thereafter. Spatial learning and memory acquisition were measured, and the data were analyzed using SMART 3.0 (Harvard Apparatus, Holliston, MA 01746, USA) [20].

### 2.6. Tissue Preparation

After the completion of the Y-maze and MWM behavioral tests, on day 9, all animals were anesthetized, their skull was opened, and the hippocampus and cortex were collected from their brains. Within the groups, the hippocampus and cortex of a subset of animals were used to assess the AChE activity; the hippocampus and cortex of the other animals were isolated and stored a –80°C for biochemical and Western blot analysis.

### 2.7. Measurement of AChE Activity

The AChE activity in the hippocampus and cortex of the mice after AD−1 small molecule, α-asarone, and tacrine treatment was measured using the spectrophotometric method developed by Karthivashan et al. [21] with some modifications. The rate of thiocholine production was determined based on the continuous reaction of thiol with 5,5-dithiobis-2-nitrobenzoate ion to produce the yellow anion of 5-thio-2-nitrobenzoic acid. The absorbance of the mixture was read at 412 nm at 30 s intervals for 5 min, and the substrate was added shortly thereafter; the percentage of inhibition was calculated.

### 2.8. Measurement of Lipid Peroxidation, GSH, SOD, Catalase, and GPx Activities

Hippocampus and cortex samples were prepared, and MDA, GSH, SOD, catalase, and GPx assays were carried out according to the manufacturer’s protocol, as previously described [21]. The absorbance of MDA, GSH, SOD, catalase, and GPx activities were measured at 410 nm, 415 nm, 490 nm, 520 nm, and 340 nm, respectively, using a UV spectrophotometer.

### 2.9. Western Blotting

Protein was isolated from the hippocampus and cortex of the mice using a 1× RIPA lysis buffer with an inhibitor of protease and phosphatase (1:1). Equal amounts of protein were subjected to 10–15% of sodium dodecyl sulphate–polyacrylamide electrophoresis and then transferred onto polyvinylidene difluoride membranes (Millipore, Bedford, MA, USA). The membranes were incubated for 1 h with a blocking reagent (3% BSA) and placed on a shaker at room temperature. Thereafter, the blots were incubated overnight at 4 °C on a rocking platform with specific primary antibodies, including anti-iNOS (ab15323, Abcam), anti-COX-2 (ab15191, Abcam), anti-BDNF (ab108319, Abcam), anti-p-CREB (#9196, Cell signaling), anti-Nrf2 (ab137550, Abcam), anti-HO-1 (#70081, Cell signaling), anti-p38 (#9212S, Cell signaling), anti-p-p38 (#9201S, Cell signaling), anti-JNK (#9253S, Cell signaling), anti-p-JNK (#9251S, Cell signaling), anti-ERK (#91012S, Cell signaling), anti-p-ERK (#9101S, Cell signaling), anti-NF-κB/p65 (F-6 #sc-8008, Santa Cruz Biotechnology), anti-p-NF-κB/pp65 (#3033S, Cell signaling), anti-IκB (#4812S, Cell signaling), anti-p-IκB (#2859S, Cell signaling), anti-caspase-3 (#H-277, Santa Cruz Biotechnology), anti-Bcl2 (#N-19, Santa Cruz Biotechnology), Bax (CD150, Santa Cruz Biotechnology), and anti-β-actin (A1978; Sigma-Aldrich), at a concentration of 1:5000. The following day, each blot was incubated at room temperature with either an anti-mouse or anti-rabbit (1:10,000) secondary antibody. The protein bands were visualized using an enhanced chemiluminescence detection system (LAS 500; GE Healthcare Bio-Sciences AB, 751 25, Uppsala, Sweden) as per the recommended protocol. Protein band intensity was performed using ImageJ software (National Institutes of Health, Bethesda, MD, USA), and the densitometric analysis was quantified using Prism v.8.0.1 software (Graph Pad, La Jolla, CA, USA).

### 2.10. Genetic Toxicity Test

Reaction chemicals, reagents, and catalysts are used in the synthesis of raw material drugs. In the process of synthesis and decomposition, impurities are present in the raw material drug. In this test, an in silico genotoxicity evaluation was conducted on the starting materials and reactants used in the synthesis. Two types of programs were used to predict genotoxicity; Derek is a model for predicting toxicity based on expertise, and Sarah is a model for predicting toxicity based on statistics. The genetic toxicity is evaluated through the complementary action of the two programs.

### 2.11. Statistical Analysis

All statistical analyses were performed using GraphPad Prism 8.0.1 (Dotmatics; La Jolla, CA, USA). The data obtained from the in vivo experiment were presented as means ± standard deviations (at least three independent experiments). We used a one-way ANOVA, followed by Duncan’s multiple comparisons test; *p* < 0.05 was considered to indicate a significant difference.

## 3. Results

### 3.1. Genotoxicity Evaluation of AD−1 Small Molecule

In the genotoxicity assessment according to the ICH M7 guidelines, 2,4,5-trimethoxybenzaldehyde and diethyl malonate were classified as class 5 (Table 1). Class 5 refers to a structure with sufficient data to prove that there is no warning structure or mutagenicity. In other words, the in silico genotoxicity was not predicted in the starting material and reactant of AD−1 small molecule. This can be used as basic data in evaluating the genotoxicity of drugs based on AD−1 small molecule.

The residual amount of the solvents used in the AD−1 small molecule synthesis process was checked using gas chromatography (Table 2). As a result of measuring the residual solvent of AD−1 small molecule, dichloromethane and hexane were detected in excess of the USP Limit. In the future, research to properly remove residual solvents in the process of mass synthesis and the development of raw material drugs is considered necessary.

### 3.2. Effect of AD−1 Small Molecule on Cognitive and memory impairments in the Scopolamine-Treated Mice

To investigate the effects of AD−1 small molecule on cognitive and behavioral impairments, we conducted Y-maze and MWM tests after the oral administration of 1 mg/kg of AD−1 small molecule for 7 days (Figure 2A). As a positive control, tacrine (10 mg/kg) was administered using the same treatment protocol. The Y-maze test showed that a scopolamine injection reduced the percentage of spontaneous alteration compared with the control treatment (*p* < 0.001); AD−1 small molecule (1 mg/kg) (*p* < 0.001), α-asarone (10 mg/kg) (*p* < 0.001), and tacrine (10 mg/kg) (*p* < 0.001) treatments significantly reduced the percentage of spontaneous alteration compared with a scopolamine injection alone (Figure 2B). However, the total number of arm entries did not appear to change between the groups, suggesting that the locomotor activity was not affected by the AD−1 small molecule (Figure 2C).

In the MWM test, the total distance and escape latency during the training period did not significantly differ between the groups (Figure 2D,E). Figure 2D,F show that a scopolamine injection significantly influenced the learning ability of the mice during acquisition (*p* < 0.001). The traveled distance before locating the platform significantly increased in the scopolamine-treated group compared to the control group (*p* < 0.001). Furthermore, the escape latency of the scopolamine-treated group also increased compared to the control group (*p* < 0.001), indicating that memory impairment was successfully induced by a scopolamine administration. In contrast, learning and memorization abilities significantly improved among the mice treated with AD−1 small molecule (*p* < 0.001). The treatments decreased the traveled distance and escape latency compared with a scopolamine injection alone (Figure 2F) (*p* < 0.001). These results confirm that the AD−1 small molecule treatment could protect the cognitive function from the scopolamine-induced memory impairment associated with AD. α-asarone (10 mg/kg) (*p* < 0.001) and tacrine (10 mg/kg) (*p* < 0.001) also exhibited similar effects on the scopolamine-induced decrease in the memory impairment and an increase in the cognitive function.

### 3.3. Effect of AD−1 Small Molecule on AChE Activity in the Hippocampus and Cortex of Scopolamine-Treated Mice

**Figure 3:**
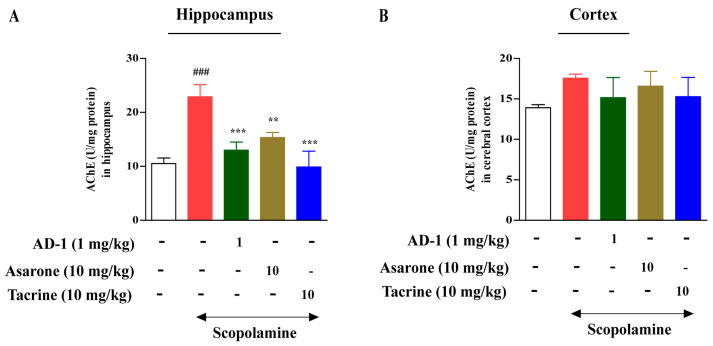
Effect of AD−1 small molecule on AChE activity in the hippocampus and cortex of scopolamine-treated mice (**A**,**B**). The data were analyzed by one-way ANOVA followed by the Tukey test and presented as mean ± SD (n = 6 per group). ^###^ *p* < 0.001 scopolamine-treated group versus control; ** *p* < 0.01 and *** *p* < 0.001 group treated with AD−1 small molecule, asarone, and tacrine/scopolamine compared to group treated with scopolamine alone. (**A**,**B**) AChE; hippocampus and cortex [F(4,10) = 23.39 and 1.87)].

As shown in Figure 3, the scopolamine treatment significantly (*p* < 0.001) increased AChE activity in the hippocampus when compared with the control group. Treatment with AD−1 small molecule (1 mg/kg) inhibited AChE activation in the hippocampus (*p* < 0.001) compared with the scopolamine-treated group (Figure 3A). α-asarone (10 mg/kg) (*p* < 0.01) and tacrine (10 mg/kg) (*p* < 0.001) also resulted in the significant inhibition of AChE activity in the hippocampus (Figure 3A). In terms of the AChE activity in the cortex (Figure 3B), no significant differences were observed among the treated groups.

### 3.4. Effect of AD−1 small molecule on Lipid Peroxidation and Antioxidant Biomarkers in the Hippocampus and Cortex of Scopolamine-Treated Mice

**Figure 4:**
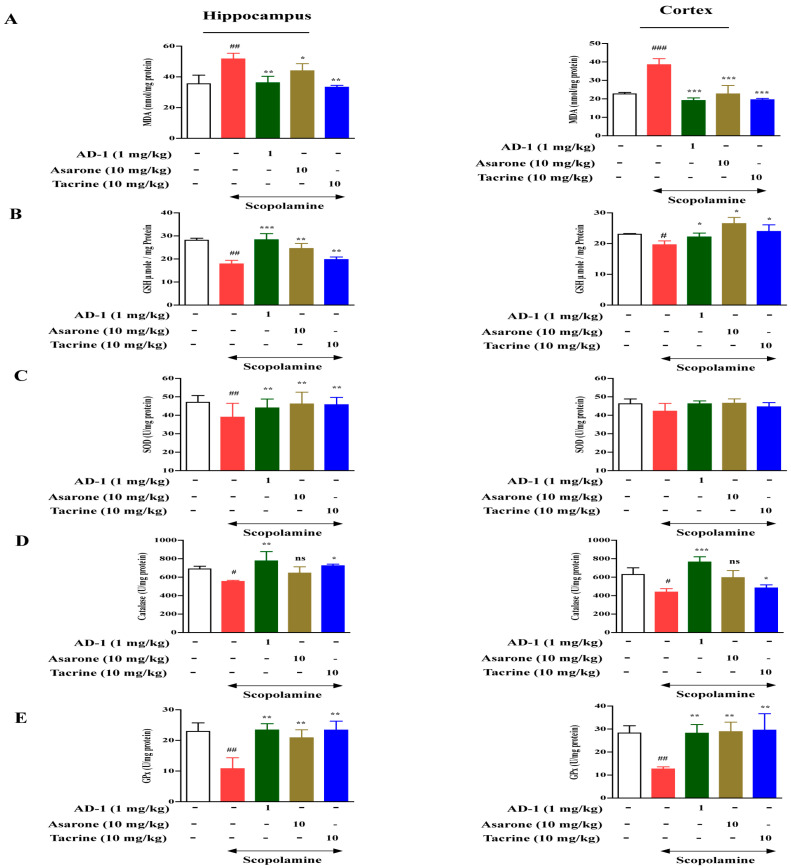
Effect of AD−1 small molecule on antioxidant status in scopolamine-treated mice hippocampus and cortex. The levels of MDA (**A**), GSH content (**B**), SOD activity (**C**), catalase activity (**D**), and GPx activity (**E**). The data are presented as mean ± SD (n = 6 per group). # *p* < 0.05, ^##^ *p* < 0.01 and ^###^ *p* < 0.001 scopolamine-treated group versus control; * *p* < 0.05, ** *p* < 0.01, and *** *p* < 0.001, ^ns^ non-significant group treated with AD−1 small molecule, asarone, and tacrine/scopolamine compared to group treated with scopolamine alone. (**A**) MDA; hippocampus and cortex [F(4,10) = 10.12 and 28.00)], (**B**) GSH; hippocampus and cortex [F(4,10) = 21.21 and 8.02)], (**C**) SOD; hippocampus and cortex [F(4,10) = 1.03 and 1.26)], (**D**) catalase; hippocampus and cortex [F(4,10) = 6.59 and 14.58)], (**E**) GPx; hippocampus and cortex [F(4,10) = 10.94 and 8.37)].

As shown in Figure 4, the MDA levels in the hippocampus and cortex were significantly increased in the scopolamine-treated group compared with the control group. However, AD−1 small molecule, α-asarone, and tacrine administration demonstrated a significant decrease in the MDA levels in the hippocampus (*p* < 0.01, *p* < 0.05, and *p* < 0.01, respectively) and cortex (*p* < 0.001) compared with the scopolamine-injected group (Figure 4A). As presented in Figure 3B, the GSH levels significantly decreased in the scopolamine-injected group compared to the control group. However, following treatment with 1 mg/kg of AD−1 small molecule, α-asarone, and tacrine, the GSH levels significantly increased in the hippocampus (*p* < 0.001, *p* < 0.01, and *p* < 0.01, respectively) and cortex (*p* < 0.05) compared with the scopolamine-treated animals (Figure 4B). Furthermore, the SOD, catalase, and GPx activities in the hippocampus and cortex were significantly decreased in the scopolamine-treated group compared with the control mice. However, the AD−1 small molecule treatment significantly restored the decreased levels of SOD (Figure 4C), catalase (Figure 4E), and GPx (Figure 4F) in the hippocampus and cortex compared with the scopolamine-treated animals. α-asarone (10 mg/kg) and tacrine (10 mg/kg) also resulted in a significant increase in the antioxidant enzyme activity in the hippocampus and cortex.

### 3.5. Effect of AD−1 Small Molecule on Nrf2 and HO-1 Expression in the Hippocampus and Cortex of Scopolamine-Treated Mice

**Figure 5:**
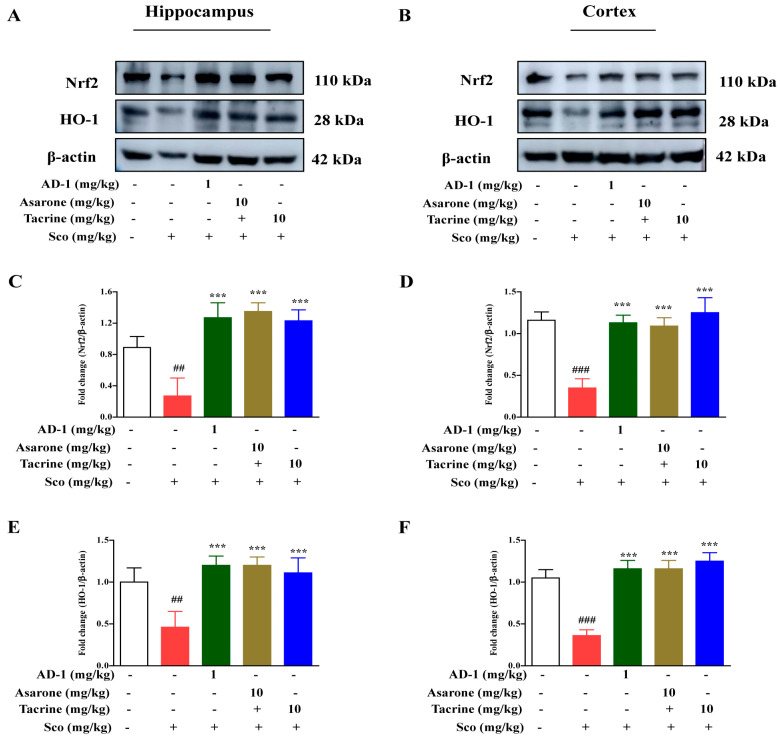
Effect of AD−1 small molecule on the protein expressions of Nrf2/HO-1 in the hippocampus and cortex of scopolamine-treated mice (**A**,**B**). The protein expression of Nrf2/HO-1 performed by Western blot analysis, and the blots were quantified using Image J software to quantify the expression of Nrf2 (**C**,**D**) and HO-1 (**E**,**F**). The data are mean ± SD (n = 3 in each group). ^##^ *p* < 0.01 and ^###^ *p* < 0.001 scopolamine-treated group versus control; *** *p* < 0.001 group treated with AD−1 small molecule, asarone, and tacrine/scopolamine compared to group treated with scopolamine alone. (**A**,**B**) Nrf2; hippocampus and cortex [F(4,10) = 20.67 and 18.06)], HO-1; hippocampus and cortex [F(4,10) = 14.42 and 18.62)].

To assess the influence of AD−1 small molecule on oxidative stress, we determine the protein expression of oxidative stress-associated protein Nrf2 and HO-1, two well established markers for antioxidant activation. Western blot analysis showed that the protein expression of Nrf2 and HO-1 were dramatically decreased in the hippocampus (*p* < 0.01) and cortex (*p* < 0.001) in scopolamine-injected mice compared with the control mice (Figure 5A,B). However, AD−1 small molecule (1 mg/kg) treatment dramatically enhanced the Nrf2 and HO-1 expression in the hippocampus (*p* < 0.001) and cortex (*p* < 0.001) compared with the scopolamine-treated animals (Figure 4A,B). α-asarone (10 mg/kg) and tacrine (10 mg/kg) also resulted in the significantly increased protein expression of Nrf2/HO-1 in the hippocampus (*p* < 0.001) and cortex (*p* < 0.001).

### 3.6. Effect of AD−1 Small Molecule on BDNF and CREB Expression in the Hippocampus and Cortex of Scopolamine-Treated Mice

**Figure 6:**
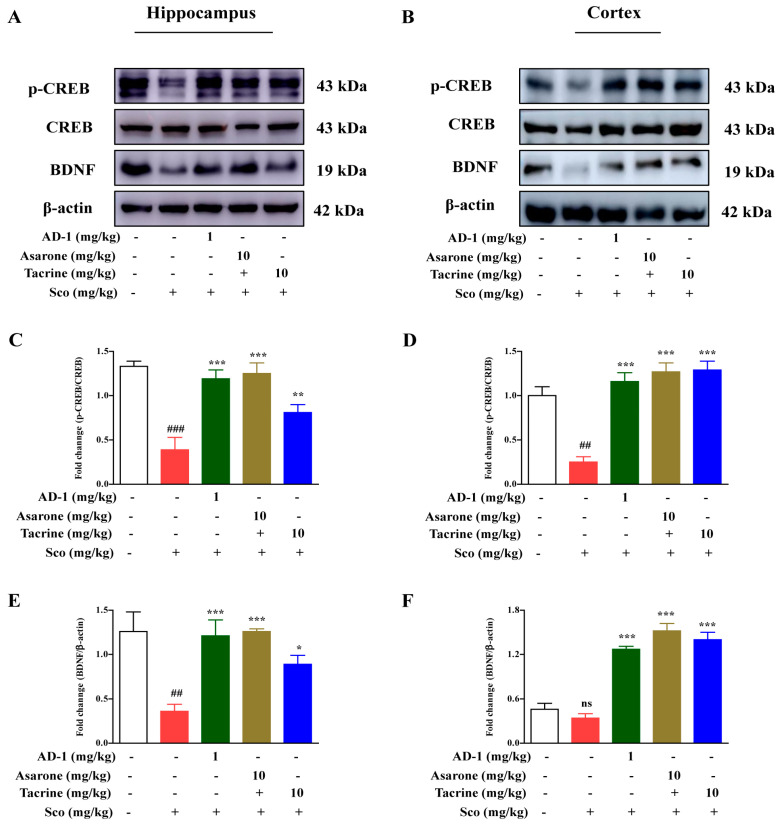
Effect of AD−1 small molecule on the protein expressions of p-CREB/BDNF in the hippocampus and cortex of scopolamine-treated mice (**A**,**B**). The protein expression of p-CREB/BDNF performed by Western blot analysis, and the blots were quantified using Image J software to quantify the expression of p-CREB (**C**,**D**) and BDNF (**E**,**F**). The data are mean ± SD (n = 3 in each group). ^##^ *p* < 0.01, ^###^ *p* < 0.001, and ^ns^ non-significant scopolamine-treated group versus control; * *p* < 0.05, ** *p* < 0.01, and *** *p* < 0.001, group treated with AD−1 small molecule, asarone, and tacrine/scopolamine compared to group treated with scopolamine alone. (**A**,**B**) p-CREB; hippocampus and cortex [F(4,10) = 21.25 and 17.87)], BDNF; hippocampus and cortex [F(4,10) = 10.97 and 46.56)].

As shown in Figure 6, the expression of BDNF and p-CREB signaling in the hippocampus and cortex decreased compared with the control group, suggesting that an injection of scopolamine could inhibit the protein expression of BDNF and p-CREB signaling (Figure 6A,B). The AD−1 small molecule (1 mg/kg) treatment could prevent a scopolamine-induced reduction in BDNF (*p* < 0.001) and p-CREB (*p* < 0.001) expression in the hippocampus and cortex compared with the scopolamine-treated group (Figure 6A,B). The α-asarone (10 mg/kg) and tacrine (10 mg/kg) administration groups also exerted the best effects, although there was a significant difference compared with the group treated with scopolamine alone.

### 3.7. Effect of AD−1 small molecule on iNOS and COX-2 expression in the Hippocampus and Cortex of Scopolamine-Treated Mice

**Figure 7:**
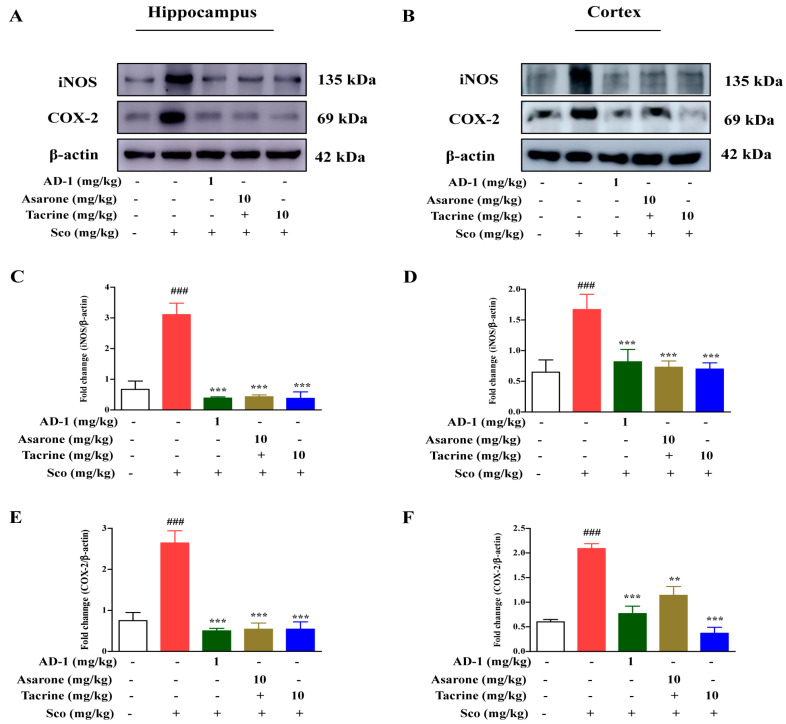
Effect of AD−1 small molecule on inflammatory mediators in the hippocampus and cortex of scopolamine-treated mice. Expression of inflammatory markers as determined by Western blot analysis (**A**,**B**). The densitometry analysis was performed using Image J software to quantify the expression of iNOS (**C**,**D**) and COX-2 (**E**,**F**). The data are mean ± SD (n = 3 in each group). ^###^ *p* < 0.001 scopolamine-treated group versus control; ** *p* < 0.01 and *** *p* < 0.001 group treated with AD−1 small molecule, asarone, and tacrine/scopolamine compared to group treated with scopolamine alone. (**A**,**B**) iNOS; hippocampus and cortex [F(4,10) = 78.81 and 23.55)], COX-2; hippocampus and cortex [F(4,10) = 17.96 and 53.70)].

The levels of activated inflammatory markers, such as iNOS and COX-2, were examined in the hippocampus and cortex from scopolamine-treated mice via Western blot analysis. The results confirmed that there was an overexpression of iNOS and COX-2 in the hippocampus and cortex in scopolamine-treated mice compared with the control mice (*p* < 0.001). However, AD−1 small molecule (1 mg/kg) demonstrated a noticeable decrease in the iNOS and COX-2 protein expression in the hippocampus and cortex compared with the scopolamine alone group (Figure 7A,B) (*p* < 0.001).

### 3.8. Effect of AD−1 small molecule on Glial Activation and NF-κB-Mediated Neuroinflammation in the Hippocampus and Cortex of Scopolamine-Treated Mice

**Figure 8:**
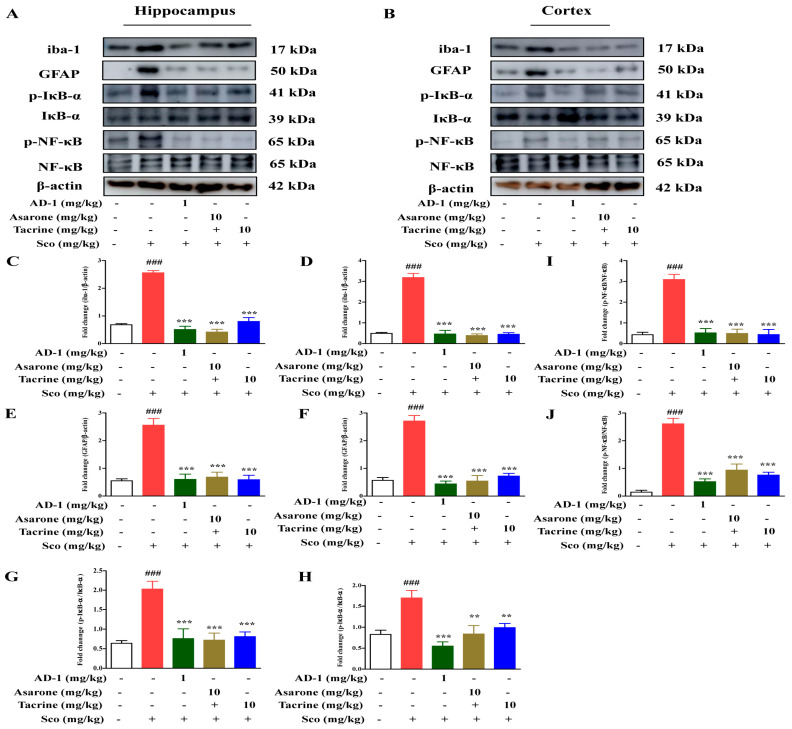
AD−1 small molecule regulated the protein of iba-1, GFAP, and p-NF-κB in the hippocampus and cortex of scopolamine-treated mice. Expression of neuroinflammatory markers as determined by Western blot analysis (**A**,**B**). The densitometry analysis was performed using Image J software to quantify the expression of iba-1 (**C**,**D**), GFAP (**E**,**F**), p-IκB-α (**G**,**H**), and p-NF-κB (**I**,**J**). The data are mean ± SD (n = 3 in each group). ^###^ *p* < 0.001 scopolamine-treated group versus control; ** *p* < 0.01 and *** *p* < 0.001 group treated with AD−1 small molecule, asarone, and tacrine/scopolamine compared to group treated with scopolamine alone. (**A**,**B**) iba-1; hippocampus and cortex [F(4,10) = 154 and 120)], GFAP; hippocampus and cortex [F(4,10) = 35.1 and 59.5)], p-IκB-α; hippocampus and cortex [F(4,10) = 63.94 and 15.16)], p-NF-κB; [F(4,10) = 64.4 and 28.6)].

Glial cells (astrocytes and microglia) play an important role in inflammation as well as inflammatory neurodegeneration because they produce several cytokines. Moreover, NF-κB overexpression may lead to the activation of several inflammatory markers that are associated with neurodegeneration. We aimed to confirm the protective effect of AD−1 small molecule against scopolamine-induced hippocampus and the cortex of iba-1 and GFAP. The results revealed that significantly elevated protein levels of GFAP and iba-1 were present in the scopolamine-injected mice (*p* < 0.001), while treatment with AD−1 small molecule reduced the levels of these proteins’ expression in the hippocampus and cortex (Figure 8A,B) (*p* < 0.001). Furthermore, the expression of NF-κB has been found to be elevated during the aging process, and a previous study has demonstrated that the brains of AD patients have revealed the presence of NF-κB in neurons and neurofibrillary tangles. The AD−1 small molecule (1 mg/kg) treatment significantly attenuated the elevated expression of p-IκB-α (*p* < 0.001) and p-NF-κB (*p* < 0.001) in the hippocampus and cortex (Figure 8A,B). The α-asarone (10 mg/kg) and tacrine (10 mg/kg) administration could significantly decrease the inflammatory protein expression in the hippocampus (*p* < 0.001) and cortex (*p* < 0.001) compared with the group treated with scopolamine alone.

### 3.9. Effect of AD−1 Small Molecule on MAPK Signaling in the Hippocampus and Cortex of Scopolamine-Treated Mice

**Figure 9:**
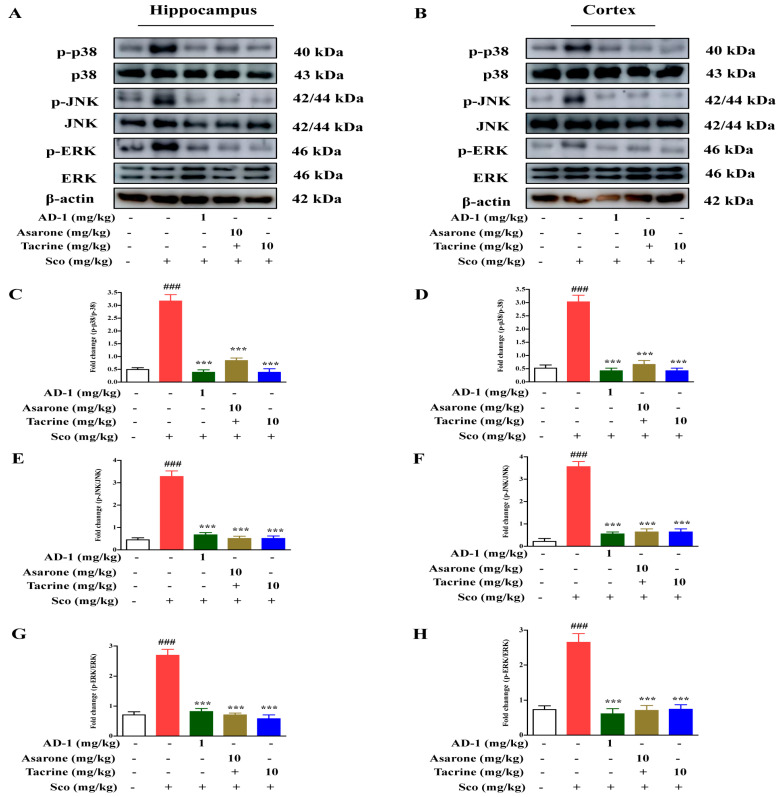
Effect of AD−1 small molecule on MAPKs protein expression in the hippocampus and cortex of scopolamine-treated mice. The MAPKs protein expression in the hippocampus and cortex were evaluated by Western blot analysis (**A**,**B**). The densitometry analysis was performed using Image J software to quantify the expression of p-p38 (**C**,**D**), p-JNK (**E**,**F**), and p-ERK (**G**,**H**). The data are mean ± SD (n = 3 in each group). ^###^ *p* < 0.001 scopolamine-treated group versus control; *** *p* < 0.001 group treated with AD−1 small molecule, asarone, and tacrine/scopolamine compared to group treated with scopolamine alone. (**A**,**B**) p-38; hippocampus and cortex [F(4,10) = 88.33 and 69.36)], p-JNK; hippocampus and cortex [F(4,10) = 98.88 and 227.4)], p-ERK; hippocampus and cortex [F(4,10) = 66.45 and 53.31)].

To confirm the beneficial effects of AD−1 small molecule, we examined the protein levels of activated MAPK signaling in scopolamine-induced mice. The Western blot data showed that scopolamine significantly increased the protein levels of p-p38 (*p* < 0.001), p-JNK (*p* < 0.001), and p-ERK (*p* < 0.001) in the hippocampus and cortex compared with the control group (Figure 9A,B). However, AD−1 small molecule alleviated the activation of p-p38 (*p* < 0.001), p-JNK (*p* < 0.001), and p-ERK (*p* < 0.001) signaling in the hippocampus and cortex compared with the scopolamine alone group, suggesting that AD−1 small molecule effectively influence the MAPK signaling pathway (Figure 9A,B). The administration of α-asarone (10 mg/kg) and tacrine (10 mg/kg) could significantly decrease the phosphorylation of MAPK signaling in the hippocampus (*p* < 0.001) and cortex (*p* < 0.001) compared with the group treated with scopolamine alone.

### 3.10. Effect of AD−1 Small Molecule on Neuronal Apoptosis in the Hippocampus and Cortex of Scopolamine-Treated Mice

**Figure 10:**
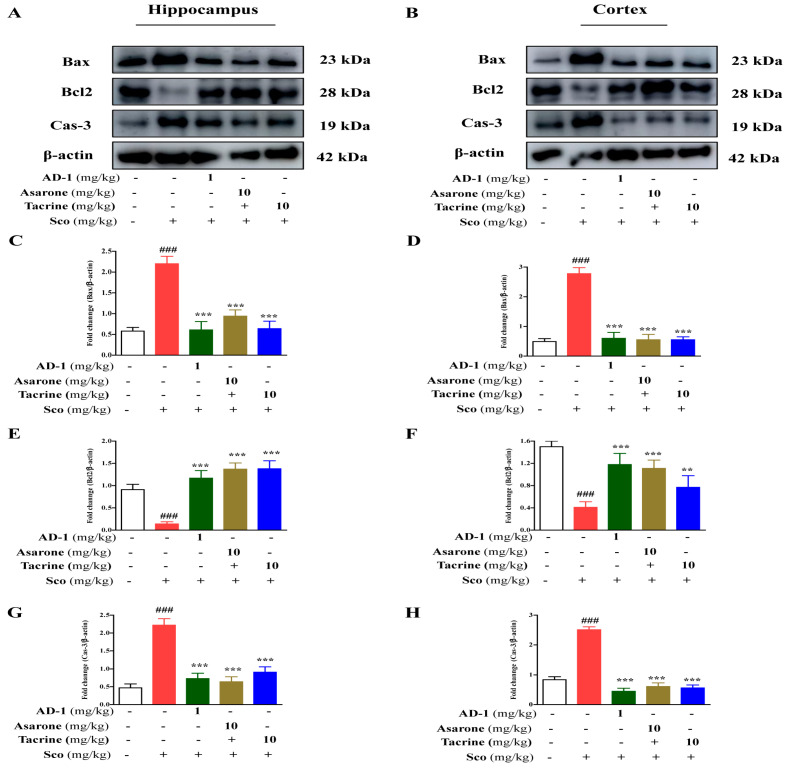
Effect of AD−1 small molecule on apoptotic markers in the scopolamine-induced hippocampus and cortex. Representative blot images showing the differential expression of apoptotic proteins (**A**,**B**). The densitometry analysis was performed using Image J software to quantify the expression of Bax (**C**,**D**), Bcl2 (**E**,**F**), and caspase-3 (**G**,**H**). The data are mean ± SD (n = 3 in each group). ^###^ *p* < 0.001 scopolamine-treated group versus control; ** *p* < 0.01 and *** *p* < 0.001 group treated with AD−1 small molecule, asarone, and tacrine/scopolamine compared to group treated with scopolamine alone. (**A**,**B**) Bax; hippocampus and cortex [F(4,10) = 27.73 and 39.05)], Bcl2; hippocampus and cortex [F(4,10) = 32.42 and 16.76)], Caspase; hippocampus and cortex [F(4,10) = 20.62 and 212.5)].

The administration of scopolamine induced neuronal apoptosis in the mice. Thus, the expression of various proapoptotic and antiapoptotic markers was examined by Western blotting. The results suggested that after the scopolamine administration, the levels of the proapoptotic protein Bax (*p* < 0.001) were significantly increased, and the administration significantly downregulated the levels of the Bcl-2 (*p* < 0.001) protein in the hippocampus and cortex compared to the control mice. As shown in the results, treatment with AD−1 small molecule and scopolamine reduced the expression of Bax (*p* < 0.001) and improved the Bcl-2 protein levels (Figure 10A,B) (*p* < 0.001). Furthermore, scopolamine treatment prompted the elevation of caspase-3 expression in the hippocampus and cortex (*p* < 0.001). Conversely, treatment with AD−1 small molecule caused a significant decrease in the expression of caspase-3 in the hippocampus and cortex of mice (Figure 10A,B) (*p* < 0.001). The administration of α-asarone (10 mg/kg) and tacrine (10 mg/kg) significantly regulated the proapoptotic and antiapoptotic markers in the hippocampus (*p* < 0.001) and cortex (*p* < 0.001) compared with the group treated with scopolamine alone.

## 4. Discussion

AD is the most common neurodegenerative disorder and leads to a gradual impairment of memory, language, problem solving, and other cognitive functions. A number of studies have extensively focused on herbal medicines to develop new therapeutic agents against AD due to the adverse effects of chemical drug treatments. Recently, we synthesized a compound, AD−1 small molecule, with neuroprotective effects from α-asarone, a safe herbal medicine. In this study, we studied the neuroprotective potential of AD−1 small molecule in in vivo scopolamine-induced memory dysfunction by evaluating the potential mechanisms involved in changes in motor activity, the spatial memory function using Y-maze and MWM tests as well as AChE activity, biochemical, and Western blot changes in the hippocampus and cerebral cortex of AD mice.

Scopolamine has been widely accepted for use in a preclinical model to study cognitive and memory impairment. In the present study, the anti-amnesic effects of AD−1 small molecule were measured in scopolamine-induced motor and cognitive deficits mice by Y-maze and MWM tasks. Our results showed that the intraperitoneal (i.p.) injection of scopolamine induced cognitive and memory impairments and that the AD−1 small molecule treatment induced a significant reduction in the cognitive impairments in scopolamine-treated C57BL/6 mice, increased the number of arm entries and higher percentage of alteration in the Y-maze test, and improved spatial learning and memory in the MWM test. These results suggest that the neuroprotective efficacy of AD−1 small molecule indicate that it would be a suitable therapeutic agent against cognitive impairments.

In order to evaluate the underlying mechanism of the memory enhancement activity of AD−1 small molecule in scopolamine-induced mice, the levels of AchE activity, biochemical, and antioxidant markers related to oxidative stress were examined. A high level of AchE activity prompts the brain to dysfunction in Ach transmission at several brain regions such as the hippocampus, cortical, and neocortical regions [22]. Thus, the inhibition of AchE activity serves as a therapeutic target for the treatment of AD. In this study, a significant rise in the hippocampus and cortex in terms of AchE activity was noted in scopolamine-administered mice. Our results showed that AD−1 small molecule could have a potential effect in terms of improving learning and memory by inhibiting the activity of AchE in the hippocampus and cortex. Moreover, accumulating evidence suggests that the induction of AchE activity by scopolamine involves a reduction in the activity of anti-oxidative enzymes and an increase in oxidative stress [2,21]. Our results were consistent with previous reports which suggested that scopolamine-treated mice lead to an imbalance of the brain’s oxidative status [2,21], as evidenced by increased MDA levels, subsequently suppressed antioxidant enzyme levels (GSH, SOD, CAT, and GPx), along with alterations in the Nrf2/HO-1 protein expression. Furthermore, the AD−1 small molecule treatment against scopolamine reduced MDA levels, markedly enhanced the antioxidant enzymes, such as GSH, SOD, CAT, and GPx, and increased the expression of the Nrf2/HO-1 protein.

CREB is a transcription factor which plays a vital role in various neuronal functions, especially the regulation of learning and memory [23]. CREB phosphorylation is responsible for transcriptional activation, resulting in the production of many gene products [23]. BDNF is one of the downstream targets of phosphorylated CREB, which plays a key role in synaptic plasticity and the cognitive function [14]. Moreover, the activation of the CREB-BDNF signaling pathway in the brain is a potential therapeutic target in treating cognitive disorders such as AD. Previous studies have reported that abnormalities in the CREB-BDNF signaling pathway are found in scopolamine-induced models of memory deficits [14,21]. In line with previous reports, we observed that the levels of CREB phosphorylation, as well as the amounts of BDNF protein, in the mouse hippocampus and cortex were down-regulated following the administration of scopolamine. Our findings are the first to demonstrate that the AD−1 small molecule treatment dramatically reversed the scopolamine-induced reduction in the phosphorylation of the CREB and BDNF protein expression in the hippocampus and cortex of the mice.

Accumulating evidence suggests that memory impairments are associated with increased neuroinflammation and microglia or astrocytes activation. A high level of inflammatory mediators, including iNOS and COX-2, play a crucial role in the pathogenesis of AD [24]. Previous studies have reported that NF-κB, a transcription factor, is overexpressed in activated glial cells, which further induces neuroinflammation by increasing the release of several inflammatory mediators [25]. The results demonstrated that scopolamine increased the protein expression of iba-1 and GFAP, while AD−1 small molecule reduced their expression. In addition, the expression of inflammatory mediators, such as iNOS and COX-2, was also increased in the scopolamine-administered group of mice, which may be associated with the induction of neuroinflammation-induced memory impairment [26]. AD−1 small molecule reduced the overexpression of iNOS and COX-2, preventing neuroinflammation and memory impairment in scopolamine-administered AD mice.

Mitogen-activated protein kinases (MAPK) play a pivotal role in regulating cell death and survival in response to stress [27]. In our study, AD−1 small molecule showed a neuroprotective effect by significantly reducing the phosphorylation of JNK and p-38 MAPK proteins compared with the scopolamine group. The activation of MAP kinases leads to apoptotic events in the cell. Several transcription factors are regulated by JNK and p-38, resulting in the enhanced expression of pro-apoptotic proteins and the reduced expression of anti-apoptotic proteins [28,29]. In line with the literature, the scopolamine-induced phosphorylation of MAPK proteins increased the ratio of Bax/Bcl-2 proteins and activated caspase-3 in mice brains compared with the normal control. The expression of these apoptotic proteins was markedly reduced in the AD−1 small molecule-treated groups compared with the scopolamine group. These results strongly indicate that AD−1 small molecule protects the neuronal cells from apoptosis in scopolamine-induced mice.

## 5. Conclusions

This study demonstrated that the neuroprotective effects of AD−1 small molecule exhibited anti-amnesic effects against scopolamine-induced memory dysfunction in mice, especially spatial memory related to the hippocampus and cortex. AD−1 small molecule may be attributed to the improvement of cholinergic transmission, the inhibition of AChE activity, oxidative stress, and the activation of the CREB-BDNF signaling pathway. In addition, AD−1 small molecule reduced the scopolamine-induced up-regulation of NF-κB/MAPK signaling and neuronal apoptosis. Our results suggest that AD−1 small molecule may be a promising candidate for use in the development of treatments for neurodegenerative disorders such as AD.

## Figures and Tables

**Table 1:** Evaluation of in silico genotoxicity of AD−1 small molecule. * Derek Nexus v.6.2.1, ** Sarah Nexus v.3.2.1. *** International council for harmonization of technical requirements for pharmaceuticals for human use (ICH) M7 guideline: “Assessment and control of DNA Reactive(mutagenic) impurities in Pharmaceuticals to limit potential carcinogenic risk”.

Chemical Name	Origin	(Q)SAR Model		Conclusion	ICH M7 *** Classification
		Derek *	Sarah **		
2,4,5-trimethoxy benzaldehyde	Starting material	Inactive	Negative	Negative	Class 5
Diethylmalonate	Reaction material	Inactive	Negative	Negative	Class 5
AD−1	-	Inactive	Negative	Negative	Class 5

**Table 2:** Residual solvents of AD−1 small molecule. * USP43, <467 > RESIDUAL SOLVENTS 4. LIMITS OF RESIDUAL SOLVENTS.

	AD−1 Residual Solvent(ppm)			
Synthetic solvent	Dichloromethane	Hexane	Ethyl acetate	Toluene
USP limit *	600	290	5000	890
Result	919	1683	581	240

## Data Availability

The original contributions presented in the study are included in the article. Further inquiries can be directed to the corresponding authors.
